# The promise of single session interventions for eating disorders: Lessons to be learned from research on digital mental health. Commentary on Schleider et al. (2023)

**DOI:** 10.1002/eat.23947

**Published:** 2023-03-29

**Authors:** Jake Linardon, Matthew Fuller‐Tyszkiewicz

**Affiliations:** ^1^ School of Psychology Deakin University 1 Gheringhap Street Geelong Victoria 3220 Australia; ^2^ Center for Social and Early Emotional Development Deakin University Burwood Victoria 3125 Australia

**Keywords:** accessible treatment, digital health, eating disorders, e‐health, personalized medicine, precision psychiatry, single session intervention

## Abstract

Schleider et al. propose that single session interventions (SSIs) could be a promising path toward catalyzing innovation in the development of accessible interventions for eating disorders (EDs). In this commentary, we contend that the arguments made by Schleider et al. raise many unresolved questions that continue to arise in the broader field. Drawing from our experiences with—and lessons learned from—developing, evaluating, and disseminating digital health interventions, we discuss four key empirical questions that should be addressed in order to realize the full potential of SSIs and other innovations in intervention delivery. These include: (i) for whom do we deliver an SSI; (ii) what are the optimal treatment mechanisms to target; (iii) what constitutes a “good” outcome; and (iv) where and how might we embed innovations like these. The SSI approach is a fruitful area of research enquiry, and we hope that this commentary generates further discussion and high‐quality, collaborative work related to improving treatment accessibility and clinical outcomes among people with EDs.

**Key Points**

- Drawing from experience in digital health, we present four questions to address to realize the potential of single session interventions (SSI).
- We include discussion around the need to understand for whom to deliver an SSI, the mechanisms of change, what a good outcome entails, and where to embed interventions like these.
- Addressing questions like these will ideally help improve treatment accessibility and outcomes among people with eating disorders.

Schleider et al. (2023) introduce the concept of single session interventions (SSIs; structured programs that intentionally involve only one visit or encounter with a clinic, provider, or platform) as a promising path toward catalyzing innovation in the development of accessible interventions for eating disorders (EDs). Schleider et al. present evidence from ~60 clinical trials supporting the efficacy of SSIs in reducing numerous mental health problems, but acknowledge that research on single‐session ED interventions is limited. An evidence‐informed process to designing SSIs is formulated, and a discussion of ways SSIs may be embedded within and outside the healthcare system is provided.

We agree with Schleider et al. that innovations in intervention delivery are sorely needed to ensure that more people affected by life‐threatening EDs receive the help that they deserve, and share the view that SSIs could help supplement (rather than replace) traditional forms of care. We also note that the arguments made by Schleider et al. raise many pertinent―yet unresolved―questions that continue to arise in the broader ED field. Here, we present four pressing empirical questions that we believe need to be addressed in order to realize the full potential of SSIs and other innovative intervention approaches. These questions are borne out of our experiences with―and lessons learned from―developing, testing, and disseminating digital interventions. Our goal is to spark further discussion, collaboration, and high‐quality research among like‐minded professionals who have the shared goal of wanting to improve client outcomes.

## 1 FOR WHOM DO WE DELIVER SSI?

While the *potential* reach for SSIs and other low intensity programs is high, it is likely that many individuals are skeptical about their use, are unwilling to utilize this form of help, or express strong preference for traditional forms of treatment. Factors like these could substantially diminish the reach and uptake low intensity interventions like SSIs, and, more importantly, may be associated with negative psychological effects (e.g., symptom deterioration, low self‐efficacy etc; Rozental et al., 2015). In our experience, this hesitancy is in part due to lack of familiarity with novel intervention approaches. Even though SSIs may be as short as 5 min, there is still potential for investment in targeted campaigns geared toward educating the intended population about the known benefits (and risks) of SSIs, addressing probable concerns and misconceptions, and instilling hope about the capabilities of SSIs. The extent to which single‐session ED interventions are in demand, along with identification of the types of individuals receptive to this form of care and development of techniques designed to enhance SSI uptake, are all worthy of further investigation. This could save time and resources by ensuring that clients are offered the best available treatment suited to their needs, preferences, and motivations.

Another challenge the field faces is identifying those client characteristics that signal a high probability of [un]successful outcomes. Treatment outcomes vary, yet we have limited understanding of those attributes that reliably predict and moderate outcomes for specific intervention modalities, intensities, and formats. Furthermore, variables once deemed as important outcome determinants have either failed to replicate in larger studies or explained only a small amount of variance in outcomes (Linardon et al., 2017). This makes it challenging to distinguish those for whom a low intensity intervention will be sufficient from those requiring more intensive services. This is important given that low intensity programs, despite their ease of widespread delivery, may worsen a subset of clients' symptoms, motivation and confidence to change, and perception of treatment (Rozental et al., 2015). In other words, it may not be wise to deliver an SSI or other low intensity program to someone who might experience potentially lasting negative effects simply because it is efficient, inexpensive, and easy to deliver. Being able to accurately predict the right therapeutic approach for each client at the commencement of treatment is critical.

Efforts to pool multiple datasets from existing clinical trials may facilitate this process by enabling application of advanced computational techniques that provide more precision to treatment allocation to ultimately enhance client outcomes. Generating such algorithms would yield important insights toward developing online triage systems capable of safely and accurately diverting some clients away from higher intensity to lower intensity services, thereby improving treatment efficiency and cost‐effectiveness. Enhancing collaboration and coordination within the ED research community would be required to expedite this process of treatment matching and sequencing.

## 2 WHAT ARE THE OPTIMAL TREATMENT MECHANISMS TO TARGET?

According to Schleider et al., SSIs should target one or more change mechanisms that lead to sustained improvements in core symptoms. This raises questions around what these change mechanisms are, and how can they be targeted in the most optimal and efficient way.

Earlier models of the client change process may prove helpful. In these conceptualizations, treatment manipulations are hypothesized to induce changes in treatment mechanisms, which in turn lead to changes in clinical symptoms (Hollon & Kriss, 1984). A treatment manipulation is any intervention technique introduced by a therapist or program. A treatment mechanism is a client process that causes change in relevant clinical symptoms. For instance, in the context of cognitive‐behavioral therapy, the regular eating schedule (treatment manipulation) is hypothesized to induce changes in dietary restraint (treatment mechanism), in turn leading to reductions in binge eating (clinical symptom). To advance understanding of critical change mechanisms, and to therefore inform the optimal design of a focused intervention like an SSI, it is necessary to know (i) whether changes in hypothesized treatment mechanisms cause reductions in relevant symptoms, and (ii) the extent to which treatment manipulations are associated with changes in the targeted treatment mechanism. Such research also necessitates longer follow‐ups than has been typically the case for SSIs.

Research isolating the effects of treatment manipulations and putative change mechanisms is limited. Some evidence suggests that prior changes in theory‐driven mechanisms (e.g., dietary restraint, shape/weight overvaluation) are associated with later symptom reduction in face‐to‐face treatment contexts (Linardon et al., 2017). However, these findings have either not replicated across different treatment centers or derive from small pilot studies. Furthermore, because putative change mechanisms were observed rather than manipulated, competing causal agents cannot be ruled out. Even less is known about what specific treatment manipulations induce change in the hypothesized mechanisms. For example, we still do not know whether dietary restraint (hypothesized mechanism) is most effectively and efficiently targeted through the techniques of self‐monitoring, regular eating, forbidden food exposure, cognitive restructuring, psychoeducation, or some specific combination of these. Due to their more narrow focus, SSIs may be ideally suited for advancing understanding of mechanisms of action.

## 3 WHAT CONSTITUTES A GOOD OUTCOME?

Schleider et al. make the case for SSIs having the potential to meaningfully impact those who use them, yet this concept of “meaningful change” is not explicitly defined. This is a broader, field‐wide challenge we wish to highlight, rather than a criticism leveled specifically at the authors. The common approach is to evaluate treatments in terms of statistical significance and effect metrics such as Cohen's d. In cases where a new treatment approach is being trialed, one might also compare this new treatment against the current gold standard. In our view, these statistical reference points are wrong‐footed, as the criteria for meaningful change is academic (statistical) rather than asking the more pressing question of whether this treatment leads to important and observable changes for those who engage.

To illustrate, let us take the reported meta‐analytic effect of 0.32 reported in Schleider et al. for SSIs. Assuming the outcome measure is normally distributed, this value suggests 87.3% overlap of score distributions between control and intervention group, a 59% superiority score indicating that a person picked from the intervention group at random would have a 59% chance of reporting a better outcome score than an individual picked at random from the control group. Further, the number needed to treat (NNT) is 9.90 indicating almost 10 participants would need to be treated in order to see one more participant in the intervention with better outcomes than control group counterparts. Under these conditions—and also factoring in the constraints on finite existing resources—would an NNT of 9.9 be considered a “best buy”?

Focus on change in predefined outcome measures makes sense in that our intention in delivering interventions is to reduce onset, severity, and duration of illness. However, near‐exclusive focus on these endpoints misses a vital opportunity to operationalize success in complementary ways that enhance our understanding of how the intervention works, which may also enhance long‐term outcomes. In our digital health work, we have begun to design our apps and evaluation studies with the user journey in mind. This journey entails key milestones from: (1) fostering positive views about the therapeutic benefit of digitally‐delivered interventions to (2) generating initial interest in signing up to use a specific digital intervention to (3) first use of the digital intervention to (4) sustained early use at the prescribed level to (5) completion of the full program and (6) mastery of techniques to address challenges as they arise in daily life.

From this user journey perspective, it is not only important to understand who is receptive to this treatment approach (as discussed above), but also whether and how we can shift preferences of those initially opposed to this form of intervention delivery. Understanding that information uptake is different from information enactment, it is also crucial to obtain data about whether, how, and under what specific circumstances individuals successfully use these prescribed therapeutic strategies in daily life. It is also vitally important to have some sense of what a positive early response looks like, and what these early indicators ultimately lead to. For instance, do we want to facilitate early positive responses because it directly signals an evolving symptom trajectory to ED remission (*SSIs as a potential one‐stop shop*), or because it will foster receptiveness to further treatment (*SSIs as a means to shift the modal number of treatment sessions from one to a higher and more efficacious number*)?

### 3.1 Where and how might we embed these innovations?

It is important to differentiate current from potential scalability of SSIs, as available literature on digital health highlights the gulf between actual and potential uptake. Our evaluations of consumer preferences highlight that consumers more often prefer longer, face‐to‐face therapies over briefer digital forms (Linardon et al., 2020). Our team also trialed a range of micro‐interventions in the hopes of enhancing uptake of resources that may be deployed in daily life to target symptoms as they arise. Qualitative feedback highlighted a key challenge of product perception, with consumers believing a single‐dose or brief program to be ineffectual, even when faced with evidence of prior efficacy and even with codesign elements built in. These and other findings highlight the need for an important piece of work on understanding consumer needs and preferences, and developing means to enhance motivation for uptake.

A stronger evidence‐base may also be needed to persuade healthcare and education providers to consider SSIs. Schleider and colleagues' observation that SSI uptake while on a treatment wait‐list enhanced likelihood of continuing to subsequent treatment is an important finding. If SSIs are to be offered as a solution to low treatment seeking, we first need to understand whether this population would use SSIs. If SSIs are to be offered while people wait for typical healthcare services, consideration needs to be given to the barriers and enablers of SSI integration with existing systems. Without a clearer sense of when and for whom SSIs are most suitable, widespread integration of SSI training within clinical training programs and roll‐out within healthcare settings seems unlikely. In Australia, where clinical placements span months, SSIs would be relatively easy to learn and administer, and may not take up a lot of time, but might not provide necessary challenge nor breadth of experiences to students. Understanding student, staff, and accrediting body perceptions of, and willingness to learn or teach SSIs and similar programs may be worthy of future investigation.

## 4 CONCLUSION

Exploration of the viability and clinical utility of SSIs for EDs are areas of research worthy of investment. In our view, there are many challenges the broader field faces and open questions that still need to be resolved before the full potential of SSIs and other easily accessible intervention formats can be realized. We hope that this commentary generates further high‐quality research dedicated toward understanding how, for whom, and through what mechanisms SSIs and other innovations in treatment delivery might work, as well as where and how they are best situated.

## AUTHOR CONTRIBUTIONS

**Jake Linardon:** Conceptualization; funding acquisition; writing – original draft; writing – review and editing. **Matthew Fuller‐Tyszkiewicz:** Conceptualization; funding acquisition; writing – original draft; writing – review and editing.

## FUNDING INFORMATION

J.L. (APP1196948) received a National Health and Medical Research Council Investigator Grant. M.F.‐T. (MRF1179321) received a National Health and Medical Research Council Medical Research future fund grant.

## CONFLICT OF INTEREST STATEMENT

The authors declare no conflict of interest.

## Data Availability

No data were used for this manuscript.
